# Targeting TDP-43 phosphorylation by Casein Kinase-1δ inhibitors: a novel strategy for the treatment of frontotemporal dementia

**DOI:** 10.1186/s13024-016-0102-7

**Published:** 2016-04-30

**Authors:** Carolina Alquezar, Irene G. Salado, Ana de la Encarnación, Daniel I. Pérez, Fermín Moreno, Carmen Gil, Adolfo López de Munain, Ana Martínez, Ángeles Martín-Requero

**Affiliations:** Department of Cellular and Molecular Medicine, Centro de Investigaciones Biológicas (CSIC), Ramiro de Maeztu 9, 28040 Madrid, Spain; Department of Chemical and Physical Biology, Centro de Investigaciones Biológicas (CSIC), Ramiro de Maeztu 9, 28040 Madrid, Spain; Neuroscience Area-Institute Biodonostia, San Sebastian, Spain; Department of Neurology, Hospital Donostia, San Sebastian, Spain; Department of Neurosciences, University of Basque Country, San Sebastián, Spain; CIBER de Enfermedades Neurodegenerativas (CIBERNED), Madrid, Spain; CIBER de Enfermedades Raras (CIBERER), Madrid, Spain

**Keywords:** FTLD-TDP, Lymphocytes, Cell proliferation, TDP-43, CK-1δ, CDK6

## Abstract

**Background:**

Mutations in the *progranulin* gene (*GRN*) are the most common cause of frontotemporal lobar degeneration with TDP-43 inclusions (FTLD-TDP). TDP-43 pathology is characterized by the hyperphosphorylation of the protein at Serine 409/410 residues. Casein kinase-1δ (CK-1δ) was reported to phosphorylate TDP-43 directly. Previous works from our laboratory described the presence of CDK6/pRb-dependent cell cycle alterations, and cytosolic accumulation of TDP-43 protein in lymphoblast from FTLD-TDP patients carriers of a loss-of function mutation in *GRN* gene (c.709-1G > A). In this work, we have investigated the effects of two brain penetrant CK-1δ inhibitors (IGS-2.7 and IGS-3.27) designed and synthetized in our laboratory on cell proliferation, TDP-43 phosphorylation and subcellular localization, as well as their effects on the known nuclear TDP-43 function repressing the expression of *CDK6.*

**Results:**

We report here that both CK-1δ inhibitors (IGS-2.7 and IGS-3.27) normalized the proliferative activity of PGRN-deficient lymphoblasts by preventing the phosphorylation of TDP-43 fragments, its nucleo-cytosol translocation and the overactivation of the CDK6/pRb cascade. Moreover, ours results show neuroprotective effects of CK-1δ inhibitors in a neuronal cell model of induced TDP-43 phosphorylation.

**Conclusions:**

Our results suggest that modulating CK-1δ activity could be considered a novel therapeutic approach for the treatment of FTLD-TDP and other TDP-43 proteinopathies.

## Background

Frontotemporal lobar degeneration (FTLD) is the primary cause of early onset dementia after Alzheimer’s disease (AD) and it is characterized by progressive alterations in behaviour, personality and language [1–3]. Mutations in *tau* (*MAPT*) and *progranulin* (*GRN*) genes and a repeat expansion in *C9orf72* are the most common genetic alterations observed in FTLD patients [4–7]. Histochemically, FTLD can be subdivided according to the major component of the protein inclusions deposited in the brain. Around 50 % of the patients can be assigned to the subgroup named FTLD-TDP, because the pathological protein present in the inclusions is TDP-43 (transactive response DNA-binding protein 43 KDa). In the most of the cases, FTLD-TDP is associated with *GRN* mutations [4, 5]. Deposition of TDP-43 has been also been detected in some patients with amyotrophic lateral sclerosis (ALS) [8], in consonance with the fact that these two diseases share some clinical and genetic features such as mutations the in *TARDBP*, *FUS* and *C9orf72* genes [9].

TDP-43 is an evolutionarily conserved nuclear protein that can bind to DNA and RNA, repress transcription, and initiate exon skipping [10]. Under physiological conditions TDP-43 is a predominantly nuclear protein. Its pathology is characterized by hyperphosphorylation, ubiquitination, cleavage of C-terminal fragments, and nucleus-to-cytoplasm translocation [8, 11], and its pathogenesis may involve both loss of normal function in the nucleus and toxic gain of function in the cytoplasm [12].

The phosphorylation of TDP-43 at tandem serines 409 and 410 characterizes all TDP-43 proteinopathy cases and therefore it is considered a hallmark of pathological TDP-43 [13, 14]. It is known that phosphorylation of site Ser 409/410 of TDP-43 leads to oligomerization and fibril formation in vitro [13]. Phosphorylation of TDP-43 may also play a role inhibiting the ubiquitin–proteasome system mediated degradation, contributing to the formation of aggregates [15]. On the other hand, the mutation of serines 409 and 410 to aspartic acid reduces the TDP-43 aggregation [16].

Casein kinases 1 and 2 (CK-1 and CK-2) were shown to phosphorylate TDP-43 in vitro [13]. However, antibodies raised against TDP-43 label in histological sections of FTLD and ALS brains show strong reactivity only for phosphorylated epitopes generated by CK-1 [13]. In addition, it was demonstrated that the products of CK-1 phosphorylation in vitro had similar electrophoretic mobility than hyperphosphorylated TDP-43 present in brain inclusions in FTLD patients [17]. Together, these observations suggest that CK-1-mediated TDP-43 phosphorylation play a role in disease pathogenesis.

CK-1 is a Ser/Thr protein kinase that is ubiquitously expressed in eukaryotic organisms [17]. At least seven isoforms (α, β, γ1 − 3, δ, and ε) and various splice variants have been characterized in different organisms [18]. Among them, CK-1δ has been determined to phosphorylate many different sites on TDP-43 *in vitro* [19]. Recently, we have developed a number of potent, very selective and brain permeable CK-1δ inhibitors. These compounds are benzothiazolyl derivatives that showed a selectivity index “S” score of 0.04 after being tested on a wide panel of more than 450 different protein kinases [20]. We have demonstrated that CK-1δ inhibition prevents TDP-43 phosphorylation in vitro decreasing its neurotoxicity in *drosophila* models [20]. The present work was undertaken to further explore the potential of these CK-1δ inhibitors to overcome main pathologic features of cells derived from FTLD-TPD patients. Our previous work highlighted the role of the CDK6/pRb pathway controlling cell fate survival/death of lymphoblasts from carriers of a loss-of-function *GRN* mutation, c.709-1G > A [21, 22]. It was suggested that an aberrant activation of this cascade could have pathogenic significance in PGRN deficiency-linked FTLD, as it is believed that unscheduled cell cycle entry underlies neuronal loss in neurodegenerative disorders [23–26]. The re-entry of quiescent neurons into the cell cycle may result in a mitotic failure and cell death [27–29]. Moreover, we found accumulated TDP-43 in the cytoplasm of these PGRN-deficient lymphoblasts [21, 30]. Therefore, it appears that these cell lines from patients, easily accessible, could represent a suitable platform to search novel disease-modifying drugs. Here, we report the effects of two brain penetrant CK-1δ inhibitors, (IGS-2.7 and IGS-3.27), in TDP-43 phosphorylation levels, cytoplasmic TDP-43 accumulation, loss of TDP-43 nuclear function, and proliferative activity of immortalized lymphocytes from FTLD-TDP patients. Both compounds were able to normalize the aberrant cell cycle control and pathological distribution of TDP-43 of PGRN deficient lymphoblasts. Furthermore, our results show a neuroprotective effect of these drugs in a neuronal model of induced TDP-43 phosphorylation. Finally, an *in vivo* pharmacokinetic study of IGS-2.7 confirms the brain penetration in mice after i.p. and oral administration. It is suggested that these drugs can be considered promising candidates for novel treatments for FTLD associated to *GRN* mutations and others pathologies in which TDP-43 is involved.

## Results

### Effects of CK-1δ inhibitors on proliferation of lymphoblasts from control and c.709-1G > A *GRN* mutation carriers individuals

**Fig. 1:**
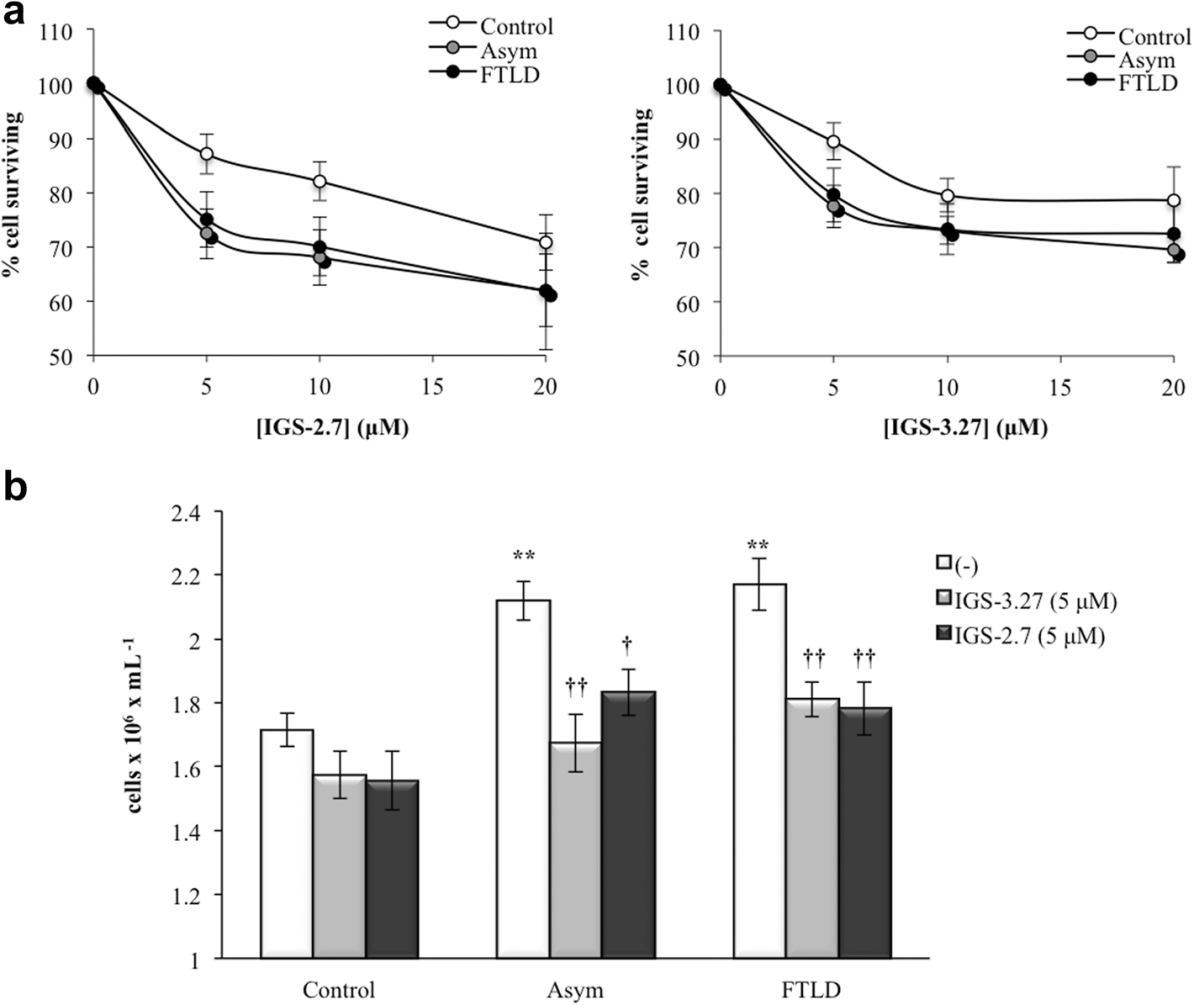
Effects of CK1-δ inhibitors on the proliferation of lymphoblasts from control and c.709-1G > A *GRN* mutation carriers individuals. Immortalized lymphocytes from control and carriers of a *GRN* mutation, asymptomatic or FTLD-TDP patients were seeded at an initial density of 1 × 10^6^ × ml^−1^ in the absence or in presence of two CK1-δ activity inhibitors, IGS-2.7 and IGS-3.27, for 72 h. **a** For the MTT assay a total of 100,000 cells per well were seeded in a 96-well plate in presence of increasing doses (0–20 μM) of both drugs. Results represent the % of cell survival of treated cells referred to untreated ones. **b** Effect of the treatment with IGS-2.7 and IGS-3.27 (5 μM) on proliferation of control and PGRN deficient lymphoblasts. Aliquots were taken for cell counting 72 h after the drug administration. Data shown are the mean ± SEM of 6 independent experiments carried out with all the cell lines used in this studio (**p < 0.01 significantly different from control cells. ††p < 0.01 significantly different from untreated cells; †*p* < 0.01 significantly different from untreated cells)

**Fig. 2:**
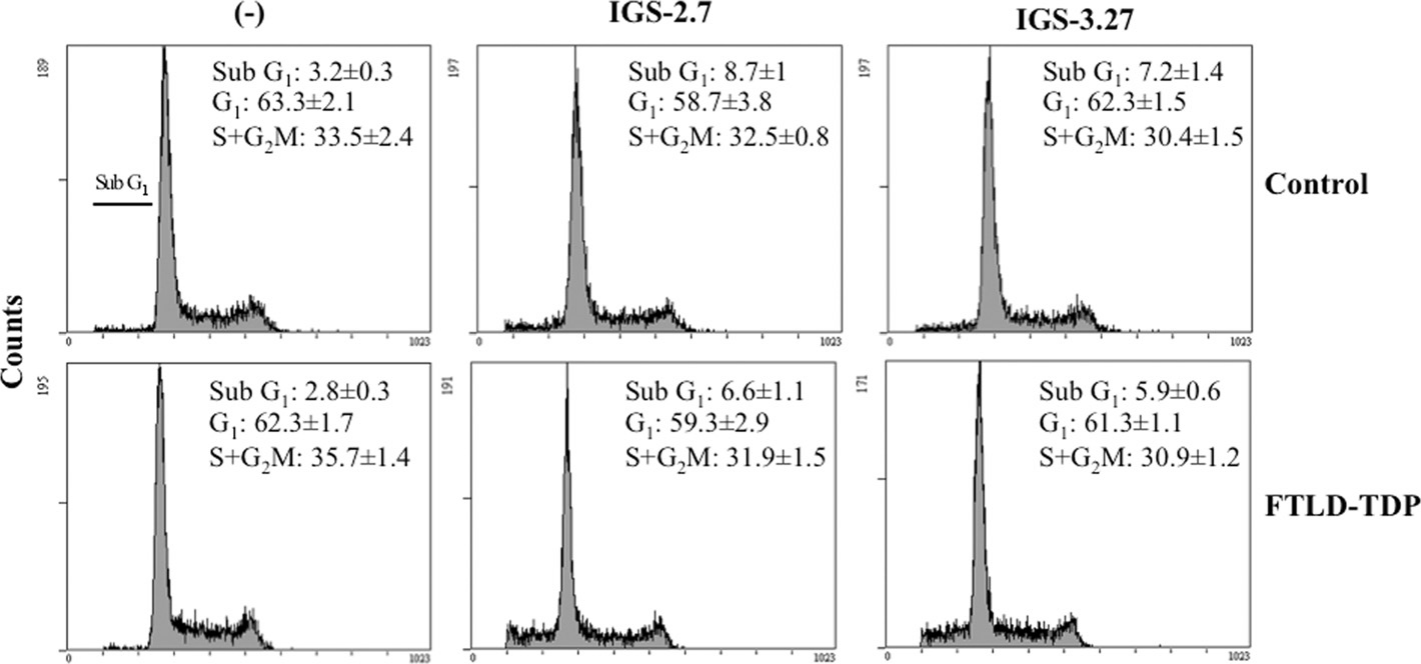
Cell cycle analysis of control and FTLD-TDP lymphoblast after treatment with CK1-δ inhibitors. Immortalized lymphocytes from control and *GRN* mutation-related FTLD individuals were seeded at an initial density of 1 × 10^6^ × ml^−1^ and cultured in RPMI medium in absence or presence of IGS-2.7 and IGS-3.27 (5 μM). 36 h after drugs addition, cells were harvested, fixed, and analyzed by flow cytometry as described under Methods. The mean ± SEM of the percentage of cells in the different cell cycle phases of four independent experiments is indicated for each condition

Previous work from this laboratory demonstrated that lymphoblasts derived from carriers of a loss-of-function *GRN* mutation (c.709-1G > A), asymptomatic or FTLD patients, show higher proliferative activity compared with control lymphoblasts, that is associated with increased levels of CDK6 protein [21, 22]. In addition, these lymphoblastoid cell lines display enhanced accumulation of cytosolic TDP-43, a hallmark of FTLD-TDP disease [22, 30]. The TDP-43 protein present in the brain deposits in FTLD-TDP patients is highly phosphorylated [8], being CK-1δ kinase involved in phosphorylation of the molecule [13, 19]. On these grounds, we sought to elucidate whether newly designed CK-1δ inhibitors are able to normalize the distinct features of PGRN deficient lymphoblasts. First, we carried out dose-response experiments to evaluate the influence of increasing concentrations of two *N*-benzothiazolyl-2-phenyl-acetamides derivatives (IGS-2.7 and IGS-3.27) on viability of lymphoblasts from control and carriers of *GRN* mutation, asymptomatic and FTLD-TDP patients. As shown in Fig. 1a, both compounds decreased cell viability, assessed by the MTT assay, in a dose-dependent manner although with higher sensitivity in lymphoblasts from PGRN deficient lymphoblasts. Further experiments were carried out at 5 μM. The proliferative activity of control and PGRN deficient lymphoblasts was measured by total cell counting after treating cells with either 5 μM IGS-2.7 or IGS-3.27 for 72 h (Fig. 1b). As is shown in Fig. 1b, the addition of IGS-2.7 and IGS-3.27 abrogated the enhanced proliferative response of PGRN deficient cells, affecting in a lesser extent the proliferation of control cells. Since the change in cell number depends on the balance between cell proliferation and cell death, we tested whether the CK-1δ inhibitors induced cell death by necrosis/apoptosis. For this purpose, we analyzed the distribution of cells in the cell cycle phases. We did not observe statistically significant changes in the proportion of subG_1_ hypodiploid cells, characteristic of apoptosis/necrosis, in control and PGRN deficient lymphoblasts after IGS-2.7 or IGS-3.27 treatment (Fig. 2). Together, these results suggest that the decreased cell number in cultures of lymphoblasts from *GRN* mutation c.709-1G > A carriers in the presence of CK-1δ inhibitors truly reflects a decrease in cell proliferation.

### Effects of CK-1δ inhibitors on TDP-43 phosphorylation

**Fig. 3:**
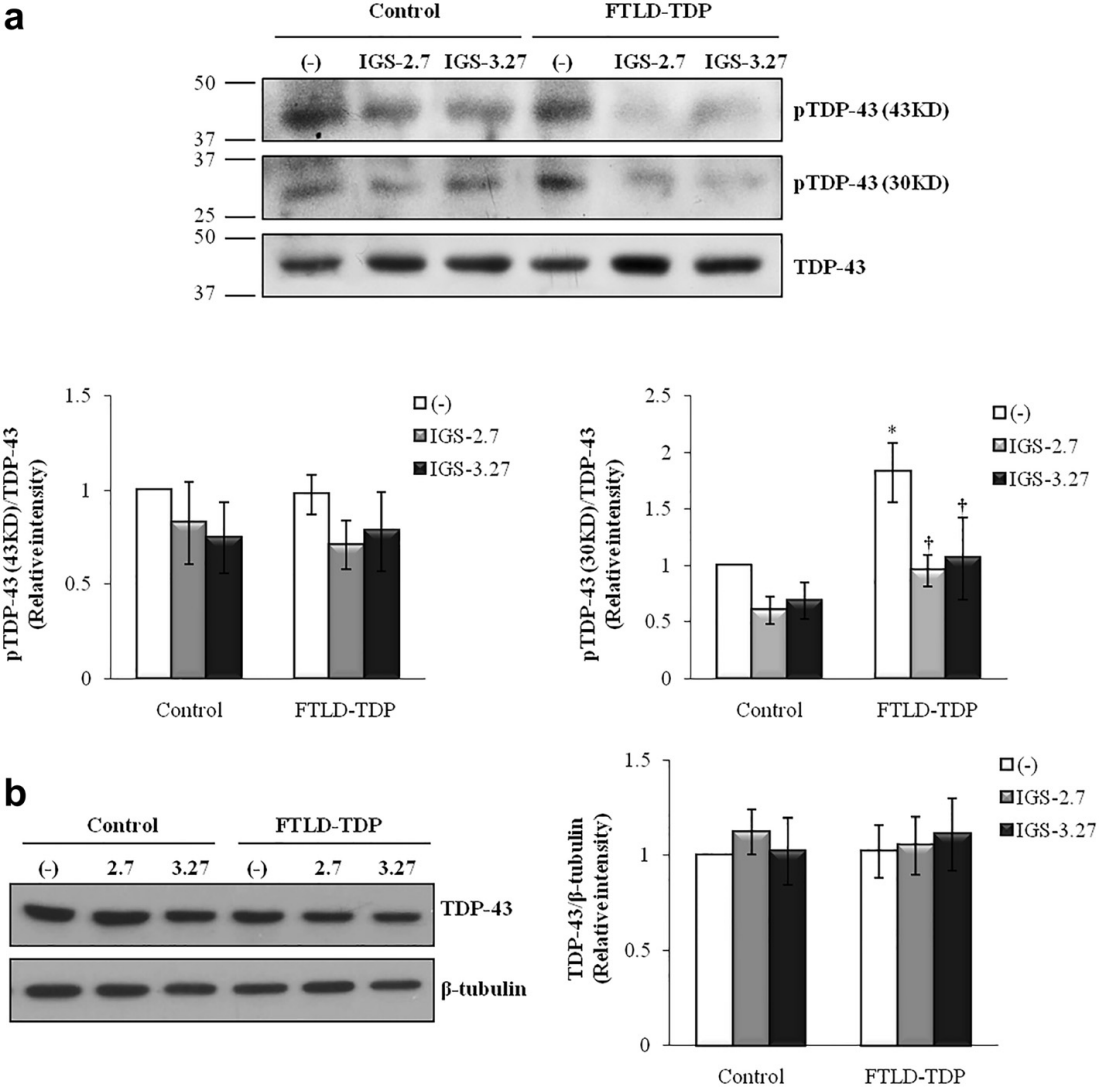
Effects of CK-1δ inhibitors on TDP-43 phosphorylation. Immortalized lymphocytes from control and *GRN* mutation-related FTLD individuals were seeded at an initial density of 1 × 10^6^ × ml^−1^ in absence or presence of IGS-2.7 and IGS-3.27 (5 μM). 24 h after drugs addition, cells were harvested and processed for Western blotting analysis. **a** Representative immunoblot showing the effect of both CK-1δ inhibitors decreasing the phosphorylation status of truncated (30KD) TDP-43 in PGRN deficient lymphoblasts. The plots bellow represent the quantifications of the bands of 43 KD (left) and 30 KD (right) of pTDP-43 normalized by Total-TDP-43. **b** The image represents the levels of total TDP-43 protein in control and FTLD-TDP patients (left panel). Quantification of the TDP-43 band normalized with the β-tubulin levels is presented in the right panel. The densitometric analyses represent the mean ± SEM of different observations carried out in four cell lines from each group (*p < 0.05 significantly different from control cells. †*p* < 0.05 significantly different from untreated cells)

The effects of IGS-2.7 and IGS-3.27 in decreasing TDP-43 phosphorylation in control and mutant *GRN* cells were assessed by Western blotting using a phospho-specific (S409/410) anti-TDP-43 antibody. Figure 3a shows a representative immunoblot. This antibody recognizes two major bands that appear in 43 KDa and 30 KDa of molecular weight. The smaller appears to be the cleaved C-terminal region. An increase in the phosphorylation levels of TPD-43 is clearly observed in the cleaved region of the protein (30 KDa) in lymphoblast from PGRN-deficient cells (Fig. 3a). Treatment with 5 μM of either IGS-2.7 or IGS-3.27 resulted in a significant decrease in the levels of phosphorylation of TDP-43 that is more evident in PGRN-deficient lymphoblasts (Fig. 3a, lower right panel). As indicated in Fig. 3b, there were not differences in total TDP-43 levels between control and mutant lymphoblasts before and after the treatment with the CK-1δ inhibitors.

### Effects of CK-1δ inhibitors on subcellular localization of TDP-43

**Fig. 4:**
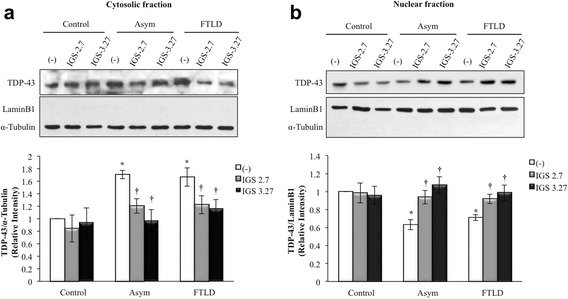
Effects of CK-1δ inhibitors on the subcellular localization of TDP-43 of control and *GRN* mutation lymphoblasts. Control and *GRN* mutation carrier lymphoblasts were seeded at an initial density of 1 × 10^6^ cells × ml^−1^ and incubated in presence or absence of IGS-2.7 and IGS-3.27 (5 μM) for 24 h. After treatment, lymphoblasts were collected and lysed to obtain the cytosolic (**a**) and nuclear (**b**) fragments that were analyzed by Western blotting. α-tubulin and LaminB1 antibodies were used as loading and purity control of the cytosolic and nuclear fractions respectively. A representative experiment is shown. Densitometric analyses represent mean ± SEM of different observations carried out in seven cell lines from each group (*p < 0.05 significantly different from control cells. †*p* < 0.05 significantly different from untreated cells)

Since it has been suggested that phosphorylation of TDP-43 may disrupt the balance between cytosolic and nuclear TDP-43 localization, we were interested in elucidating whether these CK-1δ inhibitors were able to normalize the cytosolic TDP-43 accumulation, characteristic of PGRN-deficient lymphoblasts [22, 30]. To this end, we carried out fractionation of cell extracts after the treatment with the corresponding CK-1δ inhibitor, and processed them for Western blotting with an anti-TDP-43 antibody. Our results shown that, as expected, PGRN deficient lymphoblast present increased levels of TDP-43 in the cytosolic fraction that paralleled lower nuclear content of the protein compared with lymphoblast form control individuals (Fig. 4a, b). Both treatments with IGS-2.7 and IGS-3.27 were able to decrease the levels of cytosolic TDP-43, preventing the exit of TDP-43 from the nucleus in PGRN deficient cells without affecting TDP-43 levels in control cells (Fig. 4a, b).

**Fig. 5:**
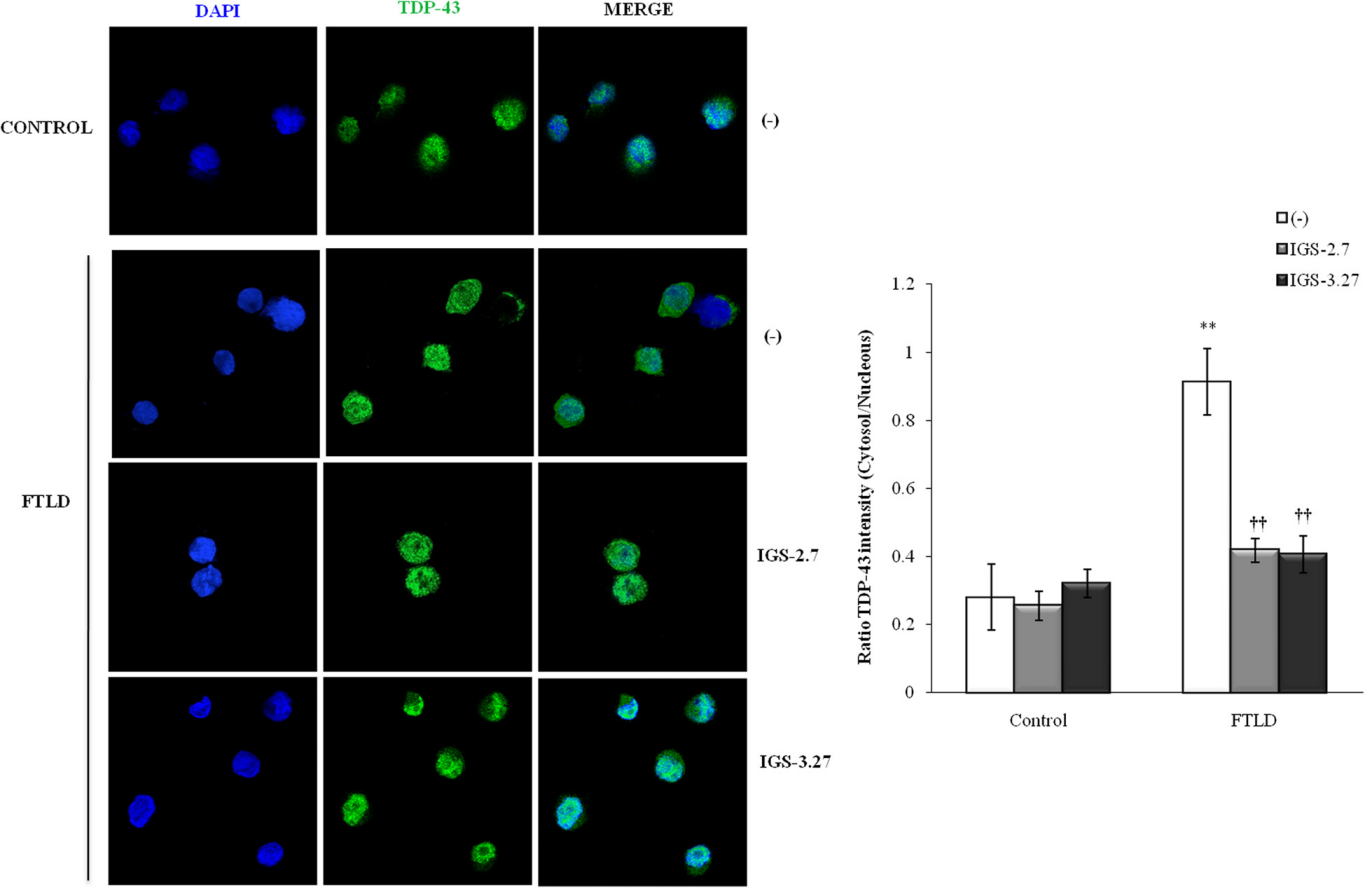
Confocal microscopy analysis of the subcellular localization of TDP-43 after CK-1δ inhibitors treatment of lymphoblast from control and FTLD-TDP patients. Lymphoblasts were seeded at 10^6^ cells × ml^−1^ and incubated in presence or absence of IGS-2.7 and IGS-3.27 (5 μM) for 24 h. TDP-43 protein localization was assessed by confocal laser scanning microscopy. Cells were stained with anti- TDP-43 antibody followed by secondary antibody labeled with Alexa Fluor 488. DAPI was included in the mounting media to stain the nucleus. Merged images show that the treatment with both drugs prevent the higher cytosolic localization of TPD-43 protein in FTLD-TDP patients. Quantitative analyses of TDP-43 redistribution are shown in the right panel. Relative fluorescence intensity of TDP-43 inside and outside nuclei was determined in 18 cells from four different fields in each condition. Magnification (63x). Values shown are the mean ± SEM. (**p < 0.01 significantly different from control cells. ††*p* < 0.01 significantly different from FTLD untreated cells)

The influence of CK-1δ inhibitors on the subcellular distribution of TDP-43 was further assessed by immunostaining using confocal microscopy. In agreement with previous work from this laboratory [22], TDP-43 predominantly localizes in the nucleus (Fig. 5), although low levels of TDP-43 are observed in the cytosol of control cells. Quantification of TDP-43 fluorescence clearly indicates that cytosolic levels of TDP-43 are increased in PGRN-deficient cells, and the addition of both IGS-2.7 and IGS-3.27 clearly favors nuclear retention of the protein (Fig. 5). Taken together, these results suggest that phosphorylation of TDP-43 plays an important role in favoring the exit of TDP-43 from the nucleus, and that CK-1δ inhibitors contribute to normalize the aberrant cytosolic TDP-43 accumulation in PGRN-deficient cells.

### Effects of CK-1δ inhibitors on CDK6 expression levels

**Fig. 6:**
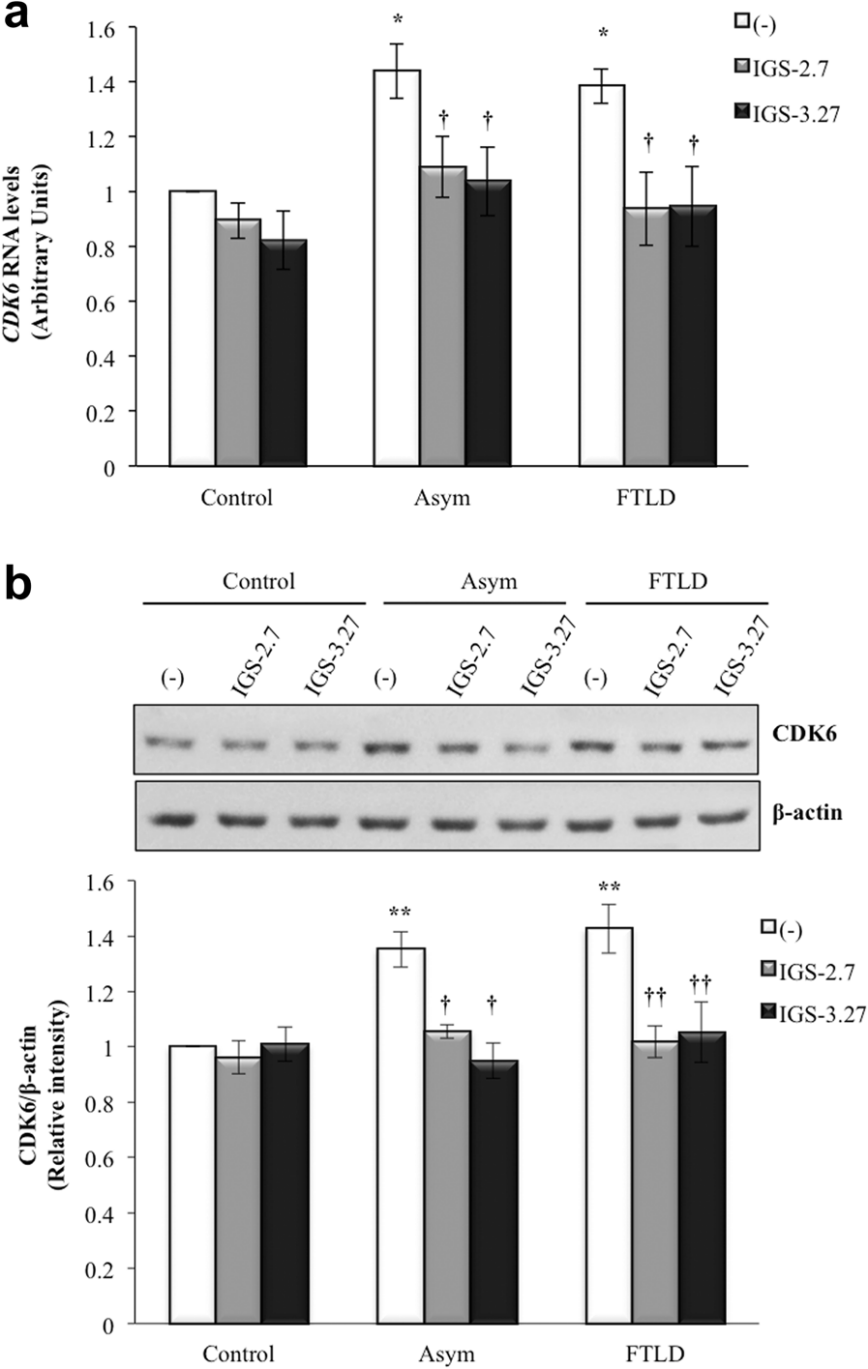
Effects of CK-1δ inhibitors on *CDK6* expression levels. Immortalized lymphocytes from control and c.709-1G > A *GRN* mutation carriers, were seeded at an initial density of 1 × 10^6^ × ml^−1^ and cultured in RPMI medium containing 10 % FBS in the absence or in the presence of IGS-2.7 and IGS-3.27 (5 μM). 24 h later cells were harvested to isolate RNA and to prepare cell lysates. **a**) *CDK6* mRNA expression levels were analyzed by quantitative RT-PCR. Data shown are the mean ± SEM of different observations using five controls, five asymptomatic and five FTLD-TDP patients. **b**) Representative immunoblot showing CDK6 protein content after drugs treatment. Densitometric measurements were performed on individual immunoblots and values indicate the mean of CDK6 levels normalized with the corresponding β-actin levels ± SEM for experiments carried out with seven different cell lines for each group (*p < 0.05 and **p < 0.01 significantly different from control cells; †*p* < 0.05 and ††*p* < 0.01 significantly different from untreated cells)

Since TDP-43 can repress *CDK6* expression [31] and CDK6/pRb pathway was found to be stimulated in lymphoblast from carriers of *GRN* mutation [22], we suggested that the increased expression of *CDK6* in PGRN deficient lymphoblast, could be the result of a loss of function of TDP-43 repressing the *CDK6* mRNA expression, secondary to changes in the nuclear content of TDP-43 [22]. For these reasons, we have evaluated the effects of IGS-2.7 and IGS-3.27 on both *CDK6* mRNA and protein levels of control and PGRN-deficient lymphoblasts. Figure 6 shows that, as expected, *CDK6* mRNA and protein levels were enhanced in PGRN-deficient lymphoblasts. Both CK-1δ inhibitors were able to decrease the expression of *CDK6* mRNA (Fig. 6a) and the protein levels (Fig. 6b).

### CK-1δ inhibitors rescue neuroblastoma cells from death induced by ethacrynic acid-mediated phosphorylation of TDP-43

**Fig. 7:**
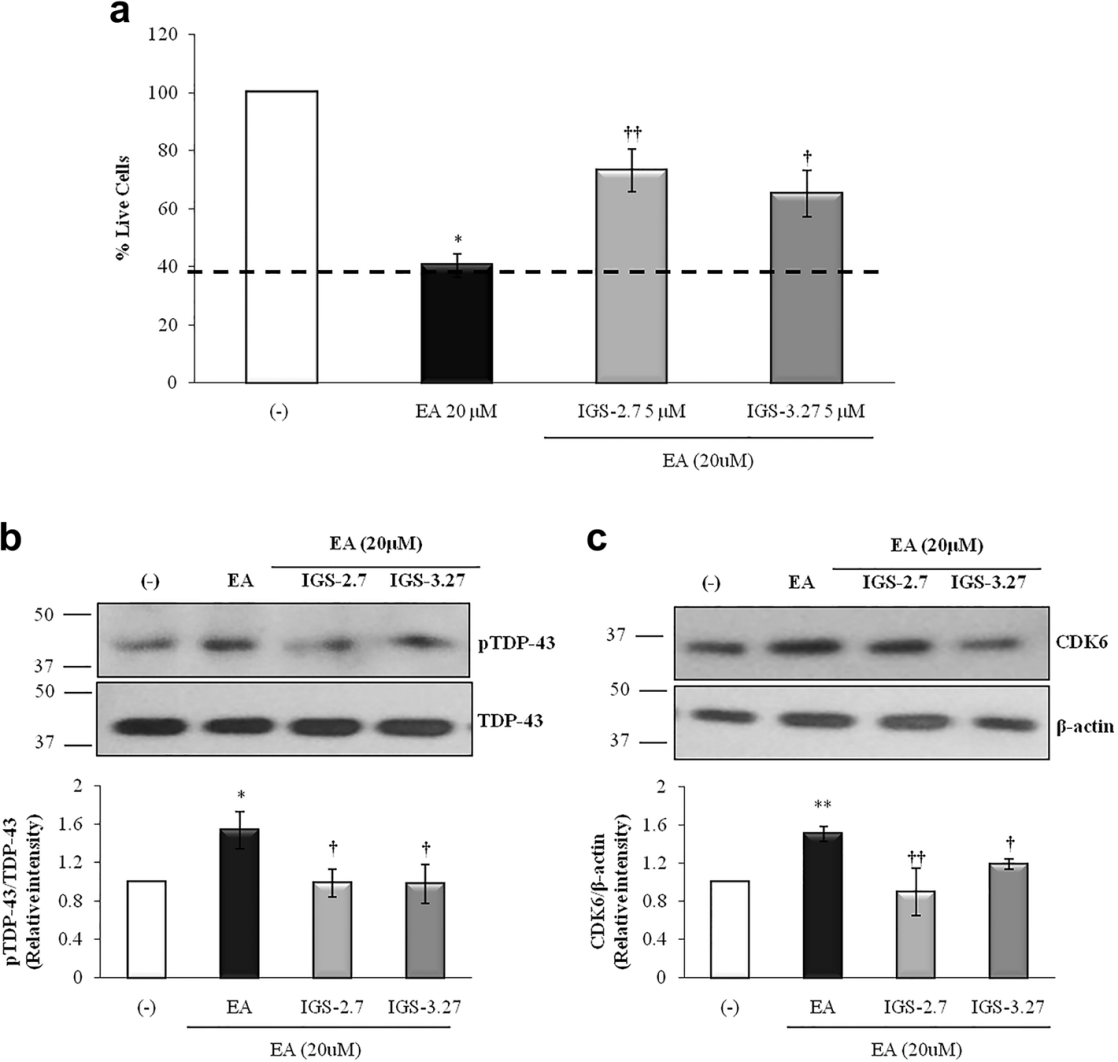
Neuroprotective effects of CK-1δ inhibitors in ethacrynic acid pre-treated SH-SY5Y neuroblastoma cells. Neuroblastoma SH-SY5Y cells were exposed to 20 μM EA for 12 h in the presence or in the absence of 5 μM of IGS-2.7 and IGS-3.27. **a** Number of viable cells after drug treatments measured by the MTT assay. Each data point represents the mean ± SEM of three replications in four different experiments (*p < 0.05 significantly different from SH-SY5Y untreated cells; †*p* < 0.05 and ††*p* < 0.01 significantly different from EA-treated cells). **b** Representative immunoblot showing the levels of pTDP-43 protein before and after drugs treatment. **c** CDK6 protein levels assessed by Western blotting. Representative immunoblot is shown. The densitometric data represent the mean ± SEM of 5 different experiments (*p < 0.05 and **p < 0.01 significantly different from SH-SY5Y untreated cells; †*p* < 0.05 and ††*p* < 0.01 significantly different from EA-treated cells)

**Fig. 8:**
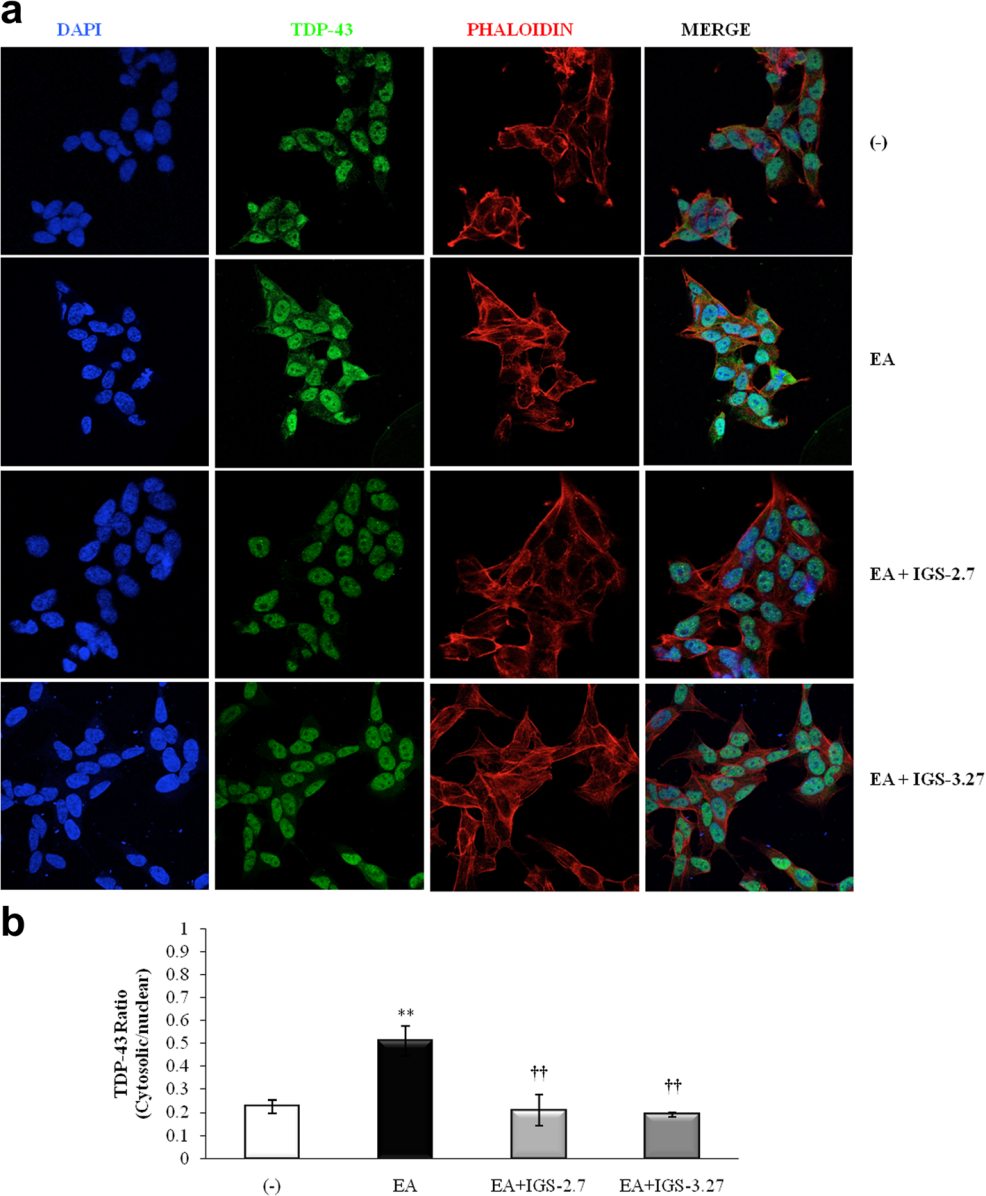
Effects of CK-1δ inhibitors on the subcellular localization of TDP-43 in ethacrynic acid pre-treated SH-SY5Yneuroblastoma cells. **a** Neuroblastoma SH-SY5Y cells were seeded in coverslips and exposed to 20 μM EA for 12 h in the presence or in the absence of 5 μM of IGS-2.7 and IGS-3.27. TDP-43 protein localization was assessed by confocal laser scanning microscopy. Cells were stained with anti-TDP-43 antibody followed by secondary antibody labeled with Alexa Fluor 488. DAPI (blue) and phalloidin (red) were used for nuclear or cytosolic staining respectively. **b** Quantitative analyses of TDP-43 redistribution. Relative fluoresecence intensity of TDP-43 staining inside and outside nuclei were determined on 30 different cells from four separate fields, in each condition. Values are the mean ± SEM (magnification 63x). (**p < 0.01 significantly different from SH-SY5Y untreated cells; ††*p* < 0.01 significantly different from EA-treated cells)

To validate the results obtained in non-neuronal cells from FTLD-TDP patients, we tested the effects of IGS-2.7 and IGS-3.27 in a neuronal cell model of induced TDP-43 phosphorylation by ethacrynic acid (EA) treatment [32] Human neuroblastoma SH-SY5Y cells were preincubated with the CK-1δ inhibitors, and then treated with 20 μM EA for 12 h. As shown in Fig. 7a, the addition of EA to the SH-SY5Y cells resulted in significant cell death, which is partially prevented by the CK-1δ inhibitors treatment. In Fig. 7b the effects of CK-1δ inhibiting the EA-dependent TDP-43 phosphorylation are shown. To elucidate whether cell cycle-related events were involved in the cell death induced by increased TDP-43 phosphorylation driven by EA, we determined the levels of CDK6 in the absence or in the presence of CK1-δ inhibitors. As it is shown in Fig. 7c, the pretreatment with CK1-δ inhibitors blunted the increase of CDK6 protein levels observed after EA addition. Together, these results add further support to the involvement of cell cycle dysregulation in neuronal fate. Finally, data in Fig. 8 suggest that phosphorylation of TDP-43 determines the subcellular localization of the protein. It is shown that both CK-1δ inhibitors, IGS-2.7 and IGS-3.27, are able to prevent cytosolic TDP-43 accumulation in EA-treated SH-SY5Y neuroblastoma cells (Fig. 8).

### The CK-1δ inhibitor IGS-2.7 shows brain penetration *in vivo*

To assess the drug-like properties of these compounds such oral absorption and brain distribution, a pharmacokinetic in vivo study of CK-1δ inhibitor IGS-2.7 in mice, was performed. IGS-2.7 was administered i.p and p.o. to mice at a dose of 2 and 10 mg.Kg^−1^, respectively. Blood samples were collected at different times and immediately after, brain samples were also collected from each mouse at the same times. Pharmacokinetic parameters were calculated using a non-compartmental analysis tool. The overall pharmacokinetic parameters summarized in Table 1 show that following a single i.p. dose administration, brain concentration were detected up to 24 h being the brain to plasma exposure ratio 2.03. Moreover, after a single oral dose administration, IGS-2.7 concentrations were detected both in plasma and brain. In that case, the brain-to-plasma exposure ratio was 3.58. These data show the good pharmacokinetic profile of CK-1δ inhibitor IGS-2.7 that crosses perfectly the blood brain barrier and can be orally administered.

**Table 1:** Pharmacokinetic parameters of the CK-1δ inhibitor IGS2-7 in plasma and brain following a single intraperitoneal (Dose: 2 mg.kg^−1^) and oral (Dose: 10 mg.kg^−1^) administration in male BALB/c mice

Route	Matrix	T_max_ (h)	C_max_ (ng.ml^−1^)	AUC_last_ (h.ng.mL^−1^)	AUC_inf_ (h.ng.mL^−1^)	MRT_last_ (h)	MRT_inf_ (h)
i.p	Plasma	0.5	224.51	1577	1689.27	6.07	7.87
	Brain^*a*^	1.0	670.56	3202.45	NR	3.40	8.64
p.o	Plasma	0.5	1120.42	6692.81	6750.19	4.61	4.82
	Brain^*a*^	2.0	3940.51	23976.84	24232.22	4.73	4.99

*NR* not reported since AUC_inf_ is 20 % greater than AUC_last_, *MRT* mean residence time

^*a*^Brain concentration and AUC expressed as ng.g^−1^ and h.ng.g^−1^ respectively

## Discussion

To date there is no specific pharmacological treatment for FTLD-TDP, being the most frequently occurring dementia in the presenile population. In the majority of the cases it is associated with mutations in the *GRN* gene. The study of PGRN haploinsufficiency and its influence altering important signaling pathways, as well as the insights into pathological processing of TDP-43 open new avenues for the identification of appropriated targets and the discovery of effective drugs.

Previously we described a cell cycle control failure in lymphoblasts from FTLD patients harboring a single pathogenic splicing mutation in the *GRN* gene (c.709-1G > A) [22]. The enhanced proliferative activity of PGRN deficient cells was accompanied by accumulation of TDP-43 in the cytosolic compartment [22, 30]. Therefore we concluded that these lymphoblastoid cell lines from FTLD-TDP patients could be a useful platform to test novel disease-modifying drugs, as they recapitulate at least two pathogenic mechanisms thought to be involved in the neurodegenerative process in FTLD, such as reactivation of cell cycle and alteration of TDP-43 subcellular distribution.

TDP-43 pathological processing includes translocation from nucleus to cytoplasm, truncation, hyperphosphorylation and ubiquitination. It is believed that abnormal phosphorylation of TDP-43 at the Ser 409/410 is a critical step in FTLD-TDP and other neurodegenerative diseases such as amyotrophic lateral sclerosis (ALS) [8, 33] Alzheimer’s disease (AD) [34], and Parkinson’s disease (PD). Since it was reported that CK-1δ is likely to be involved in TDP-43 phosphorylation *in vivo* [13], the search for specific inhibitors of this enzyme has become a challenge for the treatment of these proteinopathies [35]. Recently, we developed a number of potent CK-1δ inhibitors able to prevent TDP-43 phosphorylation in vitro and neurotoxicity in vivo [20]. Two of these benzothiazolyl amides, named IGS-2.7 and IGS-3.27 are able to cross the BBB with IC_50_ values in the nM range and great selectivity on a panel of more than 450 different kinases. Here we have evaluated the efficacy of these two drugs in normalizing the survival pattern of lymphoblasts harboring a loss-of-function *GRN* mutation. These lymphoblastoid cell lines were previously obtained from patients of FTLD-TDP and asymptomatic individuals with the mutation. For this purpose, we investigated the effects of these two drugs on the increased proliferative activity, TDP-43 phosphorylation, TDP-43 cytosolic accumulation, as well as their effects on CDK6 expression levels. These are distinct features of these PGRN-deficient lymphoblastoid cell lines [22, 30]. Moreover the potential neuroprotective role of these CK-1δ inhibitors was studied in a neuronal cell model of induced TDP-43 phosphorylation driven by ethacrynic acid treatment [32]. Our results show that IGS-2.7 and IGS-3.27 prevented the enhanced serum-mediated proliferation of PGRN deficient lymphoblasts, affecting very little the normal basal rates of proliferation in control cells. Moreover these CK-1δ inhibitors blunted the stimulated CDK6 protein and transcript levels observed in mutant lymphoblasts, thus providing an explanation for the antiproliferative effect of these drugs. At the doses used in the proliferative experiments, both IGS-2.7 and IGS-3.27 inhibited significantly the endogenous TDP-43 phosphorylation. Interestingly, the inhibition of TDP-43 phosphorylation was accompanied by reduced cytosolic TDP-43 accumulation in lymphoblasts carrying the *GRN* mutation. Taken together these results suggest that CK-1δ-mediated phosphorylation of TDP-43 may play an important role in controlling the trafficking of TDP-43 protein from the nucleus to the cytosol. This finding is in consonance with previous work showing similar effects of inhibitors of cyclin-dependent kinases (CDKs) on cytosolic TDP-43 accumulation [36]. It is worth to highlight the apparent relationship between TDP-43 phosphorylation, cytosolic TDP-43 accumulation, and cell cycle regulatory proteins. Our data showing that the PGRN deficiency-induced increased proliferative activity and CDK6 levels are accompanied by TDP-43 cytosolic accumulation, and the previous reports showing the involvement of cell division cycle 7 (CDC7) kinase on TDP-43 pathology [37] provide further support to the idea that altered cell cycle regulatory proteins may play a role in abnormal TDP-43 processing under pathological conditions.

The important question as to whether cytosolic TDP-43 accumulation implicates the gain of a new toxic function for TDP-43 and/or the concomitant reduced TDP-43 nuclear levels may represent the loss of an essential function, cannot be fully ascertained with the present data. However our results indicate that the well-known nuclear effect of TDP-43, inducing repression of *CDK6* expression [31] is blocked in PGRN-deficient lymphoblasts. These results are in consonance with a recent report indicating the loss in brains of FTD/ALS individuals, of other nuclear function of TDP-43 acting as splicing repressor of nonconserved cryptic exons [38]. Thus loss of TDP-43 nuclear functions may have pathogenic significance. Interestingly, our data demonstrate that CK-1δ inhibitors can rescue the nuclear transcriptional regulation of *CDK6* gene by preventing phosphorylation and cytosolic exportation of the TDP-43 protein.

We didn’t observe significant differences between c.709-1G > A mutation carriers, either asymptomatic or with clinical signs of dementia, regarding proliferative activity and cytosolic TDP-43 accumulation. However, it was reported that clinically asymptomatic carriers show poorer neuropsychological performance reflecting a prodromal phase of the disease [39]. Because most of the asymptomatic carriers are younger than the patients it was suggested that these alterations might be early manifestations of the disease. Thus it is tempting to speculate that CK-1δ inhibitors could hopefully be useful to slow disease progression in the early stages of disease.

Although FTLD-TDP-associated changes detected in lymphoblasts from patients may reflect those occurring in brain, it is also shown that our CK-1δ inhibitors, IGS-2.7 and IGS-3.27, were also effective preventing death in a neuronal cell model of induced TDP-43 phosphorylation with a concomitant decrease of TDP-43 phosphorylation, TDP-43 cytosolic accumulation, and blocking the increase in CDK6 levels.

## Conclusion

Our data indicate that the brain penetrant CK-1δ inhibitors are able to normalize the increased proliferation of FTLD-TDP lymphoblasts and to prevent aberrant TDP-43 cytosolic accumulation. Considering the pathogenic role of aberrant TDP-43 homeostasis and cell cycle control failure in FTLD-TDP brain, it is suggested that CK-1δ could be potentially a novel therapeutic target for the treatment of FTLD-TDP and other TDP-43 proteinopathies, with special mention to ALS, considering that a clinical, genetic and neuropathological overlap exists between FTLD-TDP and ALS. Moreover, the CK-1δ inhibitor, IGS-2.7, with excellent pharmacokinetic properties, emerges as a new drug candidate for the future treatment of neurodegenerative diseases where TDP-43 is involved.

## Methods

### Materials

All components for cell culture were obtained from Invitrogen (Barcelona, Spain). The *N*-benzothiazolyl-2-phenyl-acetamides derivatives, CK-1δ inhibitors, IGS-2.7 and IGS-3.27 were synthesized as previously described (compound 20 and 46) [20]. The chemical structure, IC_50_ values regarding CK-1δ inhibition together with effective permeability values, that predict their ability to cross the blood brain barrier (BBB) assessed by PAMPA (Parallel artificial membrane permeability assay) [40], are provided in Table 2. In the PAMPA-BBB assay the permeability of compounds IGS-2.7 and IGS-3.27 were compared with that of 10 commercial clinical drugs for experiment validation [20]. Ethacrynic acid was obtained from Sigma (Alcobendas, Spain). Antibodies against human TDP-43 (10782-2-AP) and phospho (409/410)-TDP-43 (22309-1AP) were obtained from Proteintech (Mancheser, UK). Antibodies against CDK6 (sc-177), β-actin (sc-81178) and α-tubulin (sc-23948) were obtained from Santa Cruz Biotechnologies (Santa Cruz, CA, USA) and anti-LaminB1 was purchased from Calbiochem (Billerica, MA, USA).

**Table 2:** Overview of the compounds used in this study

Compound	Structure	% inhition CK-1δ	IC_50_ [μM]	Pe (10^−6^ cm.s^−1^)	BBB prediction
IGS-2.7		>60	0.023 ± 0.002	11.3 ± 2.0	CNS+
IGS-3.27		>60	0.047 ± 0.005	4.4 ± 2.9	CNS+

The % inhibition of CK-1δ activity was determined in vitro in presence of a fixed concentration of 10 μM of the compounds. IC_50:_ half maximal concentration of both compounds inhibiting CK-1δ activity. Pe: effective permeability (data from PAMPA assay). BBB prediction. Compounds were classified as CNS+ when they present a permeability >3.74 × 10^−6^ cm · s^−1^ [20]. Values shown are the mean ± SD

### Cell lines

#### Lymphoblastic cell lines

Peripheral blood samples of all the individuals enrolled in this studio were taken after written informed consent of the patients or their relatives (demographic information is presented in Table 3) to establish the lymphoblastoid cell lines as previously described [41], by infecting peripheral blood lymphocytes with the Epstein Barr virus (EBV). All study protocols were approved by the Donostia Hospital and the Spanish Council of Higher Research Institutional Review Board and are in accordance with National and European Union Guidelines. Lymphoblastoid cells lines were grown in suspension in T flasks in an upright position, in approximately 8 ml of RPMI-1640 medium that contained 2 mM L-glutamine, 100 μg/ml streptomycin/penicillin and 10 % (v/v) fetal bovine serum (FBS) and maintained in a humidified 5 % CO_2_ incubator at 37 °C. Fluid was routinely changed every 3 days by removing the medium above the settled cells and replacing it with an equal volume of fresh medium.

**Table 3:** Characteristics of individuals enrolled in this study

		c.709-1G > A mutation carriers	
	Control *n* = 10	Asymptomatic *n* = 12	FTLD patients *n* = 7
Age (years)	51.8 ± 4.3	52.8 ± 4.3	65.3 ± 2.3
Sex, female, % (n)	50 % (5)	50 % (6)	100 % (7)
Age at onset	-	-	61 ± 0.6
Phenotype	Asymptomatic	Asymptomatic	FTD-bv; CBS
Family history, % (n)	70 % (7)	54.5 % (6)	57.1 % (4)

Control: Individuals without sing of neurological degeneration; c.709-1G > A: *GRN* mutation carriers; n: number of subjects. Values are expressed as mean ± SEM. *CBS* Cortico-basal syndrome, *FTD-bv* Frontotemporal dementia (behavior)

#### Neuronal cell culture

The human neuroblastoma SH-SY5Y cell line was propagated in Dulbecco’s Modified Eagle Medium (DMEM) containing L-glutamine (2 mM), 1 % non-essential amino acids, 1 % penicillin/streptomycin and 10 % fetal bovine serum (FBS) under humidified 5 % CO_2_. On attaining semiconfluence, cells were treated with ethacrynic acid (EA) (20 μM) for 12 h. Some cultures were pretreated for 1 h with the CK-1δ inhibitors (5 μM). After treatment, cell viability was assessed by MTT, pTDP-43 and CDK6 levels by Western blotting and the subcellular localization of TDP-43 was visualized under a confocal microscopy.

### Determination of cell proliferation, cell viability and cell cycle

Cell proliferation was determined by total cell counting, using a TC10™ Automated Cell Counter, Bio-Rad Laboratories, S.A. (Madrid, Spain). EBV-immortalized lymphocytes from control and *GRN* mutation carriers were seeded at an initial cell concentration of 1 × 10^6^ cells × mL^−1^ and enumerated everyday thereafter. Cells failing to exclude the dye were considered nonviable. Cell viability was determined by the MTT assay (3-[4,5-Dimethylthiazol-2-yl]-2,5-Diphenyltetrazolium Bromide), as previously described [42]. Cell survival was estimated as the percentage of the value of untreated controls. Cell cycle phase distribution was routinely determined by cell permeabilization followed by propidium iodide (PI) staining and flow cytometry analysis using an EPICS-XL cytofluorimeter (Coulter Científica, Móstoles, Spain).

### Immunoblotting analysis

To prepare whole-cell extract, cells were harvested, washed in PBS and then lysed in ice-cold lysis buffer as previously described [43]. To separate the cytosolic and nuclear fractions, cells were harvested, washed in PBS and then lysed in ice-cold hypotonic buffer as previously described [44]. After extraction on ice for 15 min, 0.5 % Nonidet P-40 was added and the lysed cells were centrifuged at 4,000 rpm for 10 min. Supernatants containing cytosolic proteins were separated and pellets were resuspended in hypertonic buffer to lysate the nucleus [44]. The protein content of the extracts was determined by the Pierce BCA Protein Assay kit (Thermo Scientific). 50–100 μg of protein were fractionated on a SDS polyacrylamide gel, and transferred to Poly (vinylidene) fluoride (PVDF) membranes (Millipore, Billerica, MA, USA). The membranes were then blocked with 5 % Bovine Serum Albumin (BSA) (Sigma) and incubated, overnight at 4 °C, with primary antibodies in the following concentrations, TDP-43 (1:1000); phospho-(S409/410)-TDP-43 (1:500); CDK6 (1:1000); β-actin (1:500); α-tubulin (1:1000) and Lamin B1 (1:1000). Signals from the primary antibodies were amplified using species-specific antisera conjugated with horseradish peroxidase (Bio-Rad) and detected with a chemiluminiscent substrate detection system ECL. Protein band densities were quantified using Image J software (National Institutes of Health, Bethesda, Maryland, USA) after scanning the images with a GS-800 densitometer from Bio-Rad.

### RNA preparation and quantitative real-time PCR

Total RNA was extracted from cell cultures using Trizol reagent (Invitrogen, Alcobendas, Madrid, Spain). RNA yields were quantified spectrophotometrically and RNA quality was checked by the A260/A280 ratio and on a 1.2 % agarose gel to determine the integrity of 18S and 28S ribosomal RNA. RNA was then treated with DNase I Amplification Grade (Invitrogen, Alcobendas, Madrid, Spain). One microgram was reverse transcribed with the Superscript III Reverse Transcriptase kit (Invitrogen, Alcobendas, Madrid, Spain). Quantitative real-time polymerase chain reaction (PCR) was performed in triplicates using TaqMan Universal PCR MasterMix No Amperase UNG (Applied Biosystems, Alcobendas, Madrid, Spain) reagent according to the manufacturer’s protocol. Real time quantitative PCR was performed in the Bio-Rad iQ5 system using a thermal profile of an initial 5-min melting step at 95 °C followed by 40 cycles at 95 °C for 10 s and 60 °C for 60 s. Primers were designed using the Universal ProbeLibrary for Human (Roche Applied Science, Madrid, Spain) and used at a final concentration of 20 μM. The sequences of the forward and reverse primers used are the following: for *CDK6* 5′-tgatcaactaggaaaaatcttggac-3′ and 5′-ggcaacatctctaggccagt-3′; for *β-actin*, 5’-ccaaccgcgagaagatga-3’ and 5’-ccagaggcgtacagggatag-3’. Relative messenger RNA (mRNA) levels of the genes of interest were normalized to β-actin expression using the simplified comparative threshold cycle delta-delta CT method (2^-[ΔCT *CDK6* -ΔCT *Actin*]^).

### Confocal laser scanning microscopy

Cells (1 × 10^6^ × ml^−1^ for lymphoblast and 300,000 for SH-SY5Y) were fixed for 30 min in 4 % paraformaldehyde in PBS, and blocked and permeabilized with 0.5 % TritonX-100 in PBS-0.5 % BSA for 60 min at room temperature. Then, cells were incubated overnight with anti-TDP43 polyclonal antibody. After removing the primary antibody, cells were washed with PBS and incubated with Alexa Fluor 488-conjugated anti-rabbit antibody alone or in combination with phalloidin for cytoskeleton staining. For nuclear staining, the preparations were mounted on ProLong® Gold Antifade Reagent with DAPI (Thermo Fisher) and visualized with the LEICA TCS-SP5-AOBS confocal microscope system (Heidelberg, Germany). Quantification of TDP-43 was performed using Image J software. Data is expressed as the ratio of the fluorescence intensity of cytosolic TDP-43 vs the intensity of the fluorescence of the nuclear protein.

### Pharmacokinetic *in vivo* study

A group of 48 male BALB/c mice (8–12 weeks old) weighting between 25 and 35 g following a single intraperitoneal (i.p) and oral dose (o.p) administration were used following the guidelines of the Institutional Animal Ethics Committee (IAEC). Mice were divided into two groups (Group 1: i.p. and Group 2: p.o.) with each group comprising of 24 mice. Animals in Group 1 were administered with derivative 11 solution formulation in 5 % NMP, 5 % solutol HS in normal saline intraperitoneally at a dose of 2 mg.Kg^−1^. The dosing volume administered was 10 mL.Kg^−1^. Animals in Group 2 were administered orally with derivative 11 in suspension formulation in 0.1 % Tween 80, 0.5 % NaCMC in water at a dose of 10 mg/Kg through oral gavage using a 22-G oral feeding needle. The dosing volume administered was 10 mL.Kg^−1^. Blood samples (approximately 60 mL) were collected from retro-orbital plexus under light isoflurane anesthesia from retro orbital plexus of three mice at each time point: 0.08, 0.25, 0.5, 1, 2, 4, 8 and 24 h (i.p.) and 0.25, 0.5, 1, 2, 4, 6, 8 and 24 h (p.o.). Samples were collected into labeled micro-tubes, containing 20 % K_2_EDTA solution as an anticoagulant. Plasma samples were separated from the whole blood by centrifugation at 4000 rpm for 10 min at 4 ± 2 °C and stored below −70 °C until bioanalysis. Immediately after collection of blood, brain samples were collected from each mouse at 0.08, 0.25, 0.5, 1, 2, 4, 8 and 24 h (i.p.) and 0.25, 0.5, 1, 2, 4, 6, 8 and 24 h (p.o.). Brain samples were homogenized using ice-cold phosphate buffer saline (pH 7.4) and homogenates were stored below −70 °C until analysis. All samples were processed for analysis by protein precipitation using acetonitrile. Concentrations of IGS-2.7 in mouse plasma and brain samples were determined by fit-for-purpose LC-MS/MS method using the following equipment and parameters: MS System Used**:** AB Sciex API-4000; Software Version**:** Analyst 1.5; Ion Source**:** Turbo spray; Mobile Phase**:** A**:** 0.1 % Formic acid in acetonitrile, B**:** 10 mm Ammonium formate; Flow Rate**:** 0.8 mL/min; Column Used**:** Waters Xterra, 50 × 3.0, 5 μm. The final bioanalytical method developed has a lower limit of quantification (LLOQ) of 2.01 ng/mL in plasma and 10.07 ng/mL in brain. Non-Compartmental-Analysis tool of Phoenix WinNonlin® (Version 6.3) was used to assess the pharmacokinetic parameters. Peak plasma concentrations (C_max_) and time for the peak plasma concentrations (T_max_) were the observed values. The areas under the concentration time curve (AUC_last_ and AUC_inf_) were calculated by linear trapezoidal rule. The terminal elimination rate constant, k was determined by regression analysis of the linear terminal portion of the log plasma concentration time curve. MRT was calculated by using formula as MRT = AUMC_inf_/AUC_inf_.

### Statistical analysis

Statistical analyses were performed with Graph Pad Prism 6 (La Jolla, CA, USA). All the statistical data are presented as mean ± standard error of the mean (SEM). Normality was checked with the Shapiro-Wilk test. Parametric tests were therefore used in the statistical analysis. Based on the expertise achieved on previous works [21, 22] we can expect that, with the sample size we used and the significance level we fixed, the variability within groups will be low enough and the differences between groups to detect will be high enough to ensure a statistical power above 0.9. Statistical significance was estimated by both one-way and two-way analysis of variance (ANOVA) followed by the Bonferroni’s test for multiple comparisons. A value of *p* < 0.05 was considered significant.

## Abbreviations

AD: Alzheimer’s disease
ALS: amyotrophic lateral sclerosis
BBB: blood brain barrier
*C9ORF72*: chromosome 9 open reading frame 72
CDC7: cell division cycle kinase 7
CDK6: cyclin-dependent kinase 6
CK-1δ: casein kinase 1δ
EA: ethacrynic acid
EBV: epstein barr virus
FBS: fetal bovine serum
FTD: frontotemporal dementia
FTLD: frontotemporal lobar degeneration
FUS: protein fused in sarcoma
*GRN*: granulin gene
*MAPT*: microtubule associated proteína tau
PAMPA: parallel aartificial mmembrane permeability assay
Parkinson's disease: Parkinson’s iodide
PVDF: poly (vinylidene) fluoride
TDP-43: transactive response dna-binding protein-43
